# APPLICATION OF THE CLAIM-BASED FRAILTY INDEX (CFI) TO STRUCTURED ELECTRONIC HEALTH RECORD (EHR) DATA

**DOI:** 10.1093/geroni/igae098.1260

**Published:** 2024-12-31

**Authors:** Min Ji Kwak, Caroline Schaefer, Youngran Kim, Sunyang Fu, Abhjieet Dhoble, Nahid Rianon, Holly Holmes, Dae Hyun Kim

**Affiliations:** McGovern Medical School, Houston, Texas, United States; The University of Texas Health Science Center, Houston, Texas, United States; The University of Texas Health Science Center, Houston, Texas, United States; The University of Texas Health Science Center, Houston, Texas, United States; The University of Texas Health Science Center, Houston, Texas, United States; The University of Texas Health Science Center, Houston, Texas, United States; The University of Texas Health Science Center, Houston, Texas, United States; Hebrew SeniorLife, Boston, Massachusetts, United States

## Abstract

The claim-based frailty index (CFI) was initially developed to identify frailty in Medicare claims. However, the application of CFI to structured data from an Electronic Health Record (EHR) has not been thoroughly studied. We aimed to apply CFI to structured EHR data, The Merative™ Explorys® Therapeutic Dataset Delivered, and estimate the validity of CFI compared to the 5% random sample of Medicare fee-for-service claims (2017-2018). We identified older adults (≥65 years old) with heart failure (HF) in Explorys data (n=52,295) and in Medicare claims (n=33,217). The CFI was calculated from each dataset separately. The CFI from Medicare claims showed a right-skewed distribution (mode of the CFI = 0.210) with a wider shape, whereas the CFI from Explorys data was also right-skewed but with a longer tail and had a narrower distribution (mode of the CFI = 0.186). When we assessed the prevalence of the 93 components of CFI, the components based on procedure codes were more prevalent in Medicare claims than in Explorys data. When frailty was defined as CFI ≥0.25, Explorys data identified fewer cases of frailty (18.1% vs 42.4% in Medicare claims). However, CFI from Explorys data had better discrimination (C-statistic: 0.636 vs 0.608 in Medicare claims) in predicting hospital admission or emergency room visits 1 year after the index visit. These results suggest that CFI may be implemented in EHR data and used for predicting adverse clinical events in HF patients.

